# Islet organoid as a promising model for diabetes

**DOI:** 10.1007/s13238-021-00831-0

**Published:** 2021-03-10

**Authors:** Xiaofei Zhang, Zhuo Ma, Eli Song, Tao Xu

**Affiliations:** 1 National Laboratory of Biomacromolecules, CAS Center for Excellence in Biomacromolecules, Institute of Biophysics, Chinese Academy of Sciences, Beijing, 100101 China; 2 Key Laboratory of Molecular Biophysics of the Ministry of Education, College of Life Science and Technology, Huazhong University of Science and Technology, Wuhan, 430074 China; 3 College of Life Sciences, University of Chinese Academy of Sciences, Beijing, 100049 China; 4 Guangzhou Regenerative Medicine and Health Guangdong Laboratory (Bioland Laboratory), Guangzhou, 510005 China

**Keywords:** islet organoid, diabetes, pluripotent/adult stem cell, pancreatic β cell, disease model

## Abstract

Studies on diabetes have long been hampered by a lack of authentic disease models that, ideally, should be unlimited and able to recapitulate the abnormalities involved in the development, structure, and function of human pancreatic islets under pathological conditions. Stem cell-based islet organoids faithfully recapitulate islet development *in vitro* and provide large amounts of three-dimensional functional islet biomimetic materials with a morphological structure and cellular composition similar to those of native islets. Thus, islet organoids hold great promise for modeling islet development and function, deciphering the mechanisms underlying the onset of diabetes, providing an *in vitro* human organ model for infection of viruses such as SARS-CoV-2, and contributing to drug screening and autologous islet transplantation. However, the currently established islet organoids are generally immature compared with native islets, and further efforts should be made to improve the heterogeneity and functionality of islet organoids, making it an authentic and informative disease model for diabetes. Here, we review the advances and challenges in the generation of islet organoids, focusing on human pluripotent stem cell-derived islet organoids, and the potential applications of islet organoids as disease models and regenerative therapies for diabetes.

## Introduction

Diabetes mellitus, characterized by hyperglycemia, is a prevalent metabolic disease affecting more than 400 million people worldwide that can lead to various severe complications and premature death (Saeedi et al., 2019). Type 1 diabetes (T1D) is an autoimmune disease that results in the dysfunction of β cells. Type 2 diabetes (T2D), the most common form in adults, is characterized by peripheral insulin resistance and relatively insufficient insulin secretion (Ashcroft and Rorsman, 2012). In addition, an increasing number of rare monogenic diabetes, including maturity-onset diabetes of the young (MODY) and neonatal diabetes (ND), which result from mutations in a single gene involved in pancreatic β cell development or function, have been identified (Ashcroft and Rorsman, 2012; Bishay and Greenfield, 2016). Unfortunately, both the pathogenesis of diabetes and effective therapeutic regimens remain largely unclear.

The pancreatic islet is an essential endocrine organ that secretes hormones for blood glucose homeostasis. It comprises five endocrine cell types, namely, ~60% insulin (INS)-producing β cells, ~30% glucagon (GCG)-producing α cells, ~10% somatostatin (SST)-producing δ cells, a low percentage of pancreatic polypeptide (PPY)-producing γ/PP cells, very few ghrelin (GHRL)-producing ε cells (Brissova et al., 2005; Cabrera et al., 2006), and an abundance of supporting cells (e.g., endothelial cells, macrophages, and nerve cells) (Mattsson, 2005; Aamodt and Powers, 2017). During development, islets originate from separate dorsal and ventral pancreatic buds, which then merge and expand through cell proliferation (Pictet et al., 1972; Shih et al., 2013). Simultaneously, NGN3^+^ cells delaminate from the developing pancreatic ducts to initiate islet development (Rukstalis and Habener, 2007; Gouzi et al., 2011; Shih et al., 2013), which is a delicate process that is precisely regulated by a series of transcription factors, including PDX1, NKX2.2, NKX6.1, and PAX6 (Oliver-Krasinski and Stoffers, 2008; Jennings et al., 2015; Youn et al., 2016). Unfortunately, the morphogenesis, signaling pathways and key factors involved in islet development remain largely unknown, resulting from interspecies difference, scarcity of human donors, and ethical considerations concerning human embryo research.

Organoid is a cutting-edge technology providing new models for developmental biology and disease research (Lancaster and Knoblich, 2014; Fatehullah et al., 2016; Dutta et al., 2017; Schutgens and Clevers, 2020). Organoids are three-dimensional (3D) cultures derived from stem cells, either pluripotent stem cells (PSCs) or organ-restricted adult stem cells (ASCs) and can mimic specific organ functions (Lancaster and Knoblich, 2014). Compared with animal models, organoids can be of human origin and thus can obviate the need to extrapolate discoveries from model animals to humans; compared with organ models derived directly from humans, organoids are more readily available and can be personalized. In clinical applications, organoids can serve as personalized high-throughput drug screening platforms with which to evaluate potential therapeutic efficacies, and as new sources for organ transplants due to their functionality (Fatehullah et al., 2016; Dutta et al., 2017; Schutgens and Clevers, 2020). However, current organoid systems have many problems, including limited cell compositions, insufficient maturity, incomplete function, and poor vascularization.

Because islets are closely involved in the occurrence and development of diabetes, islet organoids have attracted increasing attention. Pancreatic β cells are considered the most essential components of islets; thus, previous studies paid more attention to the generation of β cells from stem cells, termed stem cell-derived β (sc-β) cells (Pagliuca et al., 2014). Recently, rapid increasing progress has been made in sc-β cell generation and islet organoid model development. Here, we review the advances and challenges in establishing islet organoids and discuss their potential application.

## State-of-the-Art Strategies to Establish Islet Organoids

The mystery of human pancreas development has been partially uncovered. Some key signaling pathways, including the BMP, NOTCH, and canonical and noncanonical WNT pathways, and a few regulatory genes, including NKX2.2, NEUROD1, and ISL1, have been characterized (reviewed in (Jennings et al., 2015; Nair and Hebrok, 2015; Petersen et al., 2018; Yu and Xu, 2020)). Based on current knowledge about the mechanism of human islet development, a series of studies have developed various recipes combining panels of growth factors and small molecules to sequentially manipulate the signaling pathways to guide the commitment of hPSCs to a β cell fate (D’Amour et al., 2006; Kroon et al., 2008; Rezania et al., 2012). However, in these previous studies, the immaturity of sc-β cells, as demonstrated by the poor glucose responsiveness, polyhormonal signatures, and preferential commitment of the polyhormonal cells to α instead of β cells, has been reported (Kelly et al., 2011; Basford et al., 2012; Rezania et al., 2013; Bruin et al., 2014; Hrvatin et al., 2014). Currently, functional maturation relies mostly on *in vivo* transplantation (Rezania et al., 2013, 2014; Augsornworawat et al., 2020), but the underlying mechanisms remain unknown. Considerable efforts have been made to derive functional islet organoids *in vitro*.

### Optimization of recipes to obtain functional β-like cells

**Figure 1:**
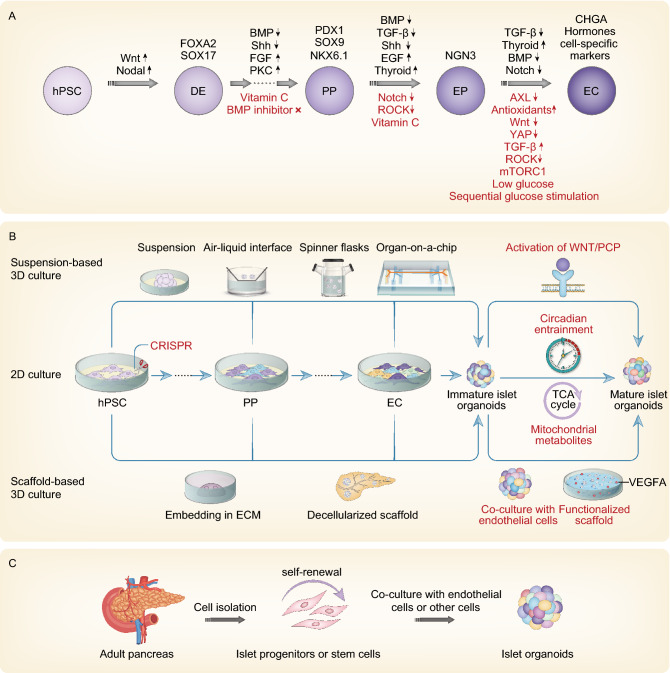
**State-of-the-art strategies to establish islet organoids**. (A) Signaling pathways typically manipulated to induce differentiation of sc-β cells. Generally, hPSCs are sequentially differentiated to definitive endoderm (DE), pancreatic progenitors (PP), endocrine precursors (EP) and endocrine cells (EC), as shown by markers of each stage. The pathways in black are commonly manipulated in the most widely used protocols (Pagliuca et al., 2014; Rezania et al., 2014; Russ et al., 2015; Nair et al., 2019), and the pathways in red are specifically reported to facilitate endocrine specification (Ghazizadeh et al., 2017; Rosado-Olivieri et al., 2019; Sharon et al., 2019; Velazco-Cruz et al., 2019; Helman et al., 2020; Hogrebe et al., 2020). (B) Strategies currently leveraged to establish 3D islet organoids. Strategies in black are used to establish 3D islet organoids, relying on either suspension or scaffold culture. 3D culture starting from different time points has been reported. Strategies in red are applied to improve the maturity of 3D islet organoids, focusing mainly on improving vascularization and recovering metabolic defects of the immature islet organoids. (C) Strategies for ASC-derived islet organoids. Islet progenitors can be isolated from the adult pancreas and then form islet organoids with endothelial cells or other cells

Recently, several groups have reported refined recipes to generate sc-β cells or accomplish islet regeneration *in vitro*. The design of these recipes relied mainly on current knowledge of pancreas development to *in vitro* mimic the natural microenvironment corresponding to specific developmental stages; therefore, most protocols share certain induction pathways, although disparities exist (summarized in Fig. 1A). Pagliuca et al. systematically tested >150 combinations of >70 compounds to formulate a 6-step protocol, which generated ~33% sc-β cells resembling primary β cells at the molecular, ultrastructural, and functional levels (Pagliuca et al., 2014). These sc-β cells expressed certain canonical β cell marker genes, including PDX1 and ZNT8, and possessed both developing and mature crystallized insulin granules, where normal insulin processing occurs (Pagliuca et al., 2014). Functionally, these cells repeatedly increased the intracellular Ca^2+^ and secreted insulin upon sequential glucose changes *in vitro* and rapidly restored euglycemia in a diabetic mouse model after transplantation, closely resembling the native islets (Pagliuca et al., 2014). As determined by multiomics analysis, three other major cell types in addition to sc-β cells existed among the final induction products, namely, α-like cells, an unexpected population of enterochromaffin cells, and SOX9^+^ pancreatic progenitors, which tend to generate exocrine cells upon further induction (Veres et al., 2019). Multiomics analysis further delineated the process of *in vitro* islet specification and identified two sequential lineage bifurcations, which lay at the initiation points of endocrine cell and β cell formation (Sharon et al., 2019; Veres et al., 2019; Alvarez-Dominguez et al., 2020). Each of the two bifurcations led to a dramatic decrease in induction efficiency and an increase in induction product heterogeneity (Sharon et al., 2019; Veres et al., 2019).

The immaturity of the sc-β cells was attributed partially to the premature induction of early precursors due to the premature expression of NGN3 in the early stages (Johansson et al., 2007). Rezania et al. introduced vitamin C during induction of the pancreatic endoderm to suppress precocious NGN3 expression and the downstream targets, NEUROD1 and NKX2.2 (Rezania et al., 2014). Vitamin C also regulates extracellular matrix (ECM) production and increases cell confluency (Choi et al., 2008). With addition of vitamin C, a population containing approximately 50% insulin^+^/NKX6.1^+^ and KCl-responding cells appeared after β cell induction; however, these cells failed to respond to high glucose concentrations, indicating their immaturity and the requirement for additional maturation steps (Rezania et al., 2014). Further screening of compounds capable of inducing MAFA, a marker of mature β cells, formulated a 7-step protocol. With this updated protocol, the majority of endocrine cells in the final products were β-like cells, ~5%–10% of which rapidly increased the cytosolic Ca^2+^ concentration upon glucose challenge. Although significant glucose stimulated insulin secretion (GSIS) was not observed, the β-like cells slowly accumulated insulin upon glucose responsiveness (Rezania et al., 2014).

The role of vitamin C in suppressing NGN3 expression is somewhat cell-specific. Russ et al. showed that NGN3 transcripts were not reduced in other cell lines upon vitamin C treatment (Russ et al., 2015). They omitted BMP inhibitor treatment during the induction of pancreatic progenitors to prevent premature endocrine commitment, instead treating progenitors with the BMP inhibitor noggin to induce endocrine precursors, allowing spontaneous differentiation into ~20% monohormonal glucose-responding β-like cells in basal medium (Russ et al., 2015). However, omission of noggin during the early pancreatic progenitor step was recently found to induce the expression of CDX2, a marker of intestinal development, at the expense of losing NKX6.1 expression and the ability for further differentiation into endocrine cells (Wesolowska-Andersen et al., 2020). However, different cell lines were employed in these studies; therefore, the action of noggin could be a cell-specific effect, as also observed with other factors, including TPB or PdBU for PKC activation, and dorsomorphin or noggin for BMP inhibition (Kunisada et al., 2012; Rezania et al., 2012; Pagliuca et al., 2014; Nostro et al., 2015). Most recently, Hogrebe et al. developed a two-dimensional (2D) protocol using latrunculin A to depolymerize the cytoskeleton during endocrine induction and strongly improve the *in vitro* and *in vivo* function of sc-β cells (Hogrebe et al., 2020).

In addition to adjusting the combinations of growth factors and small molecules, decreasing the exposure time to the progenitor-inducing cocktail yielded fewer polyhormonal cells and more NKX6.1^+^ multipotent progenitors (Nostro et al., 2015). Intriguingly, increased induction efficiency was observed by halving the cell density after the endodermal stage and extending the progenitor-inducing time (Memon et al., 2018). These results indicate that the efficiency of sc-β cell differentiation from hPSCs is mysteriously correlated with cell density, induction recipe, and cell lines.

### Strategies to establish 3D islet organoids

In addition to elaborate chemical recipes, the role of 3D microenvironments for *in vitro* islet development has been extensively investigated. Islets are densely packed 3D architectures with specific cell distribution patterns in which cell-cell interactions have significant roles in regulating cell fate specification and islet function (Roscioni et al., 2016). These roles were ignored in early attempts to establish β-like cells *in vitro*, which partially accounted for the immaturity of the derived β-like cells due to diminished growth factor gradients. Spontaneous clustering was observed when hPSC-derived endodermal cells were replaced with a quartered density (Memon et al., 2018) or when monolayer-cultured hPSCs were induced toward the posterior foregut on Matrigel-coated dishes (Broda et al., 2019). The former generated clusters containing unusual PDX1^−^/NKX6.1^+^ progenitors, and the latter have not been used for islet organoids.

#### Suspension-based 3D culture

The self-organization capacity of hPSCs has been widely employed to aggregate dissociated hPSCs into 3D embryonic bodies (EBs) with variable diameters dependent on the culture system (Fig. 1B). Pagliuca et al. utilized a stir plate to apply a constant rotation to hPSCs cultured in spinner flasks to generate ~100–200-μm cell clusters. Morphologically and functionally, these clusters resembled native human islets; however, few non-β endocrine cells were detected compared with those in human islets (Pagliuca et al., 2014). Dissociation and reclustering of islet organoids in the final stage have been reported to further improve functionality (Velazco-Cruz et al., 2019).

Microfluidic devices offer a more sophisticated suspension-based strategy. Microfluidic perfusable systems continuously deliver induction signals or nutrients while removing metabolic wastes. Furthermore, perfusion enables the application of mechanical cues, such as physiologically relevant flow-induced shear stress. Compared with the products obtained under static culture conditions, perfusable system-generated islet organoids have increased cell viability, elevated expression of mature endocrine cell markers, and significantly improved islet function (Tao et al., 2019).

Although 3D architecture plays a pivotal role in maturing islet organoids, the appropriate stage at which to apply 3D culture remains debatable. Compared with 3D suspension culture, 2D planar culture prevented pancreatic progenitors from prematurely expressing NGN3 but inhibited their further differentiation toward the endocrine lineage, indicating the significance of timing in 3D culture initiation (Hogrebe et al., 2020). Initiation of 3D culture at different stages has been reported. Inspired by the aggregated distribution of PDX1^+^/NKX6.1^+^ pancreatic progenitors in monolayer culture (Toyoda et al., 2015; Mamidi et al., 2018), starting suspension culture from progenitors increased the proportion of PDX1^+^/NKX6.1^+^ cells and the expression levels of PDX1 (Toyoda et al., 2015). The transfer of planar pancreatic endodermal cells to an air-liquid interface allowed apical-basal polarity and generated ~1–2-mm clusters with enhanced NGN3, INS, GCG and SST expression (Rezania et al., 2014). Direct evidence showing the impact of the 3D structure on islet organoid maturation was provided by comparing 2D sc-β cells and their clustered counterparts. A static suspension culture of dissociated sc-β cells generated ~50–150-μm endocrine cell clusters (ECCs) with significantly augmented coexpression of PDX1/NKX6.1 and PDX1/GLUT1; reduced expression of MAFB, a marker of endocrine progenitors; and improved functional maturation (Kim et al., 2016). The ECCs contained all endocrine cell types except α cells; however, cell heterogeneity has not been promoted by clustering, as shown by decrease of PPY and GCG expression (Kim et al., 2016). Nair et al. further examined the metabolic mechanisms underlying clustering-facilitated β cell maturation and revealed the role of clustering-promoted mitochondrial oxidative respiration in the metabolic maturation of β cells (Nair et al., 2019).

#### Scaffold-based 3D culture

Instead of relying on self-organization to establish 3D structures, other strategies incorporated ECM components as scaffolds to promote 3D structure formation and cell-matrix interactions (Fig. 1B). Dissociated mouse embryonic pancreatic progenitors can survive in 2D culture, but only in Matrigel organoids can be formed, highlighting the role of cell-matrix interactions in cell fate specification (Greggio et al., 2013). 3D islet organoids built within a scaffold combining collagen and Matrigel (C-M scaffold) yielded α, β, γ and PP cells (Wang et al., 2017). The β-like cells expressed elevated levels of INS, GLUT2 and MAFA and harbored 12-fold more insulin granules than their 2D cultured counterparts. In addition to facilitating late-stage maturation of endocrine cells, the C-M scaffold also provides a favorable microenvironment for endocrine commitment in the early developmental processes, as manifested by the reduced expression of HNF6 (restricted in exocrine cells in the native pancreas) in 3D pancreatic progenitors compared with planar counterparts (Wang et al., 2017). Candiello et al. devised a novel hydrogel-based platform called “Amikagel” by polymerizing amikacin hydrate and polyethyleneglycol diglycidyl ether (PEGDE), which facilitated the self-aggregation of hESC-derived pancreatic progenitor cells and their coaggregation with supporting endothelial cells (Candiello et al., 2018). Amikagel-produced 3D cell aggregates had increased populations of cells coexpressing NKX6.1/PDX1, increased expression of insulin/C-peptide, and enhanced glucose responsiveness (Candiello et al., 2018). Islet organoids cultured in a microporous scaffold showed improved control over islet organoid size and cell-cell interactions. These ~250–425-µm islet organoids had more mature marker expression and performed better in GSIS than their counterparts in suspension culture (Youngblood et al., 2019).

Using a decellularized pancreas as a scaffold is another elaborate and effective strategy to provide an intact biomimetic ECM microenvironment for supporting the differentiation and maturation of sc-β cells. This strategy includes the perfusion of decellularization reagents to completely remove cells residing in the pancreas followed by perfusion of a cell suspension to recellularize the scaffolds. The scaffold retains biochemical signals with ECM proteins and can mimic biophysical signals, such as shear stress, during recellularization. Mouse iPSC-derived β-like cells have been perfused into decellularized scaffolds and matured into functional β-like cells (Wan et al., 2017). Decellularized ECM hydrogels offer another more flexible microenvironment, in which the ECM composition can be manipulated and the embedding of cells is more convenient than recellularization. Native islets cultured in decellularized pancreatic ECM exhibited improved glucose responsiveness and enhanced viability of both islets and islet-resident endothelial cells (Jiang et al., 2019).

Proteomic analysis of decellularized rat pancreas revealed that collagen V was a crucial component in the islet niche (Bi et al., 2020). Suspension cultures of hPSCs in collagen V-augmented Matrigel-coated dishes generated more mature islet cells (Bi et al., 2020). Notably, in addition to biochemical signals guiding cell fate determination, the ECM components surrounding the developing cells are also in continuous flux to regulate cell differentiation to different stages. Enrichment of certain ECM components, such as laminin, promoted bipotent pancreatic progenitors to commit to endocrine cell specification, while exposure to other ECM components led to duct cell differentiation (Mamidi et al., 2018), implying that stage-specific scaffolds may facilitate endocrine differentiation *in vitro* and improve the induction efficiency.

### Innovative strategies facilitating functional maturation of islet organoids

In addition to optimization of the induction protocol and establishment of 3D structures, instructive strategies derived from the current understanding of the islet developmental process have facilitated the maturation of sc-β cells.

#### Vascularized islet organoids

*In vivo* maturation of islet organoids or hPSC-derived pancreatic progenitors typically generates islet organoids that are morphologically and functionally similar to native islets (Rezania et al., 2013, 2014; Augsornworawat et al., 2020). One of the key disparities between the features of islet organoids derived from *in vitro* environments and those derived from *in vivo* environments is their vascular networks, which are abundant in native islets and transplanted islet organoids but scarce in *in vitro*-derived products (Ranjan et al., 2009; Eberhard et al., 2010; Sneddon et al., 2018). Islets are considered one of the most highly vascularized organs due to their endocrine features. Therefore, vascularization is vital for islet development and endocrine function, and lack of vascular networks reduces not only the fidelity of islet organoids but also their viability during *in vitro* culture because of the insufficient nutrient and oxygen supply to deeply buried cells. Many strategies have attempted *in vitro* vascularization by coculturing endothelial cells with endocrine cells. Taniguchi’s group reported that coculturing of cell lines, native tissue fragments, and iPSC spheroids with human umbilical vein endothelial cells (HUVECs) and mesenchymal stem cells (MSCs) in Matrigel enabled the formation of vascularized islet organoids (Fig. 1B) (Takahashi et al., 2018a; Takahashi et al., 2018b). Gene expression patterns of the vascularized islet organoids reflected native islets better than those of non-vascularized islets (Takahashi et al., 2018a). Furthermore, a vascular-inductive bed was established by functionalizing the bioactive polyethylene glycol (PEG) hydrogel with vascular endothelial growth factor A (VEGFA), which is widely believed to be secreted by early β cells during embryonic development to recruit endothelial cells and promote the formation and expansion of the vasculature (Phelps et al., 2013). This protocol facilitated intraislet vasculature formation and significantly improved the functional performance of islets embedded in this bed and delivered to the small bowel mesentery in a diabetic mouse model (Phelps et al., 2013).

#### Other strategies

Core circadian clock activators have been predicted to facilitate maturation of the epigenomic dynamics of islet organoids (Alvarez-Dominguez et al., 2020). Daily circadian rhythms in islet organoids tuned, but did not elicit, glucose responsiveness by increasing the glucose threshold of GSIS (Alvarez-Dominguez et al., 2020). Defective GSIS but normal KCl responsiveness of β-like cells have been demonstrated in various studies. A possible reason for this impaired glucose sensing and GSIS is reduced anaplerotic cycling stemming from reduced GAPDH and PGK activity (Davis et al., 2020). The use of cell-permeable intermediate metabolites downstream of these two enzymes successfully improved islet function (Davis et al., 2020).

Gene manipulation has also been considered for the maturation of islet organoids. Inducible knockout of *LIN28B* after definitive endoderm formation significantly improved GSIS of the derived islet organoids, but the expression of mature islet markers was not significantly upregulated (Zhou et al., 2020). Overexpression of ERRγ, an important regulator of mitochondrial oxidative ATP biosynthesis, increased the glucose responsiveness of β-like cells *in vitro* and *in vivo* (Yoshihara et al., 2016).

Noncanonical WNT signaling was reported to participate in pancreas specification and islet maturation (Rodriguez-Seguel et al., 2013; Yoshihara et al., 2020). Correspondingly, treatment of immature islet organoids with WNT4, an activator of noncanonical WNT signaling, significantly increased mitochondrial content and oxidative metabolism and improved GSIS *in vitro*, without affecting cell fate determination (Yoshihara et al., 2020). Enrichment of the CD177^+^ cell subpopulations from induced definitive endoderm, in which WNT/PCP signaling was specifically activated, generated a higher percentage of MAFA^+^ mature sc-β cells with elevated glucose responsiveness (Mahaddalkar et al., 2020).

### Islet organoids derived from ASCs and other sources

ASCs residing in adult tissues have the capacity for self-renewal and differentiation under certain conditions, and they are considered another potential source for organoids (Clevers, 2016). In contrast to PSC-derived organoids, ASC-derived organoids have the identity of a given organ (Fig. 1C) (Habener, 2004) and are able to avoid tumorigenicity in potential clinical applications. There is a long-standing debate regarding whether the adult pancreas contains stem or progenitor cells. Several particular cell groups have been proposed as pancreatic progenitor or progenitor-like cells, which have the ability to differentiate into insulin-secreting cells *in vitro,* including Nestin-positive cells isolated from mouse pancreas (Wei et al., 2012), cells with high aldehyde dehydrogenase enzyme activity sorted from human fetal and adult pancreas (Loomans et al., 2018; Oakie et al., 2018), and P2RY1- and ALK3-positive cells sorted from human pancreatic ducts (Qadir et al., 2018; Qadir et al., 2020). Recently, a new population of Procr^+^ endocrine progenitors was identified from adult mouse islets (Wang et al., 2020). Procr^+^ progenitors can generate functional endocrine cells *in vitro* and *in vivo*, and islet organoids derived from Procr^+^ cells are capable of reversing hyperglycemia in a mouse model of streptozotocin-induced T1D (Wang et al., 2020). Additionally, non-β endocrine cells, pancreatic exocrine cells, and even nonpancreatic cells such as intestinal enteroendocrine cells have been demonstrated to transdifferentiate into insulin-secreting cells by reprogramming in some cases (Thorel et al., 2010; Chen et al., 2014; Lemper et al., 2015). However, genetic lineage-tracing studies indicated that the exocrine compartment was unlikely to contribute to the formation of endocrine cells *in vivo* (Aguayo-Mazzucato and Bonner-Weir, 2018; Dominguez-Bendala et al., 2019). The current dominant view is that the replenishment of adult pancreatic β cells relies principally on their self-duplication instead of potential pancreatic stem cell differentiation (Dor et al., 2004).

## APPLICATIONS OF ISLET ORGANOIDS

**Figure 2:**
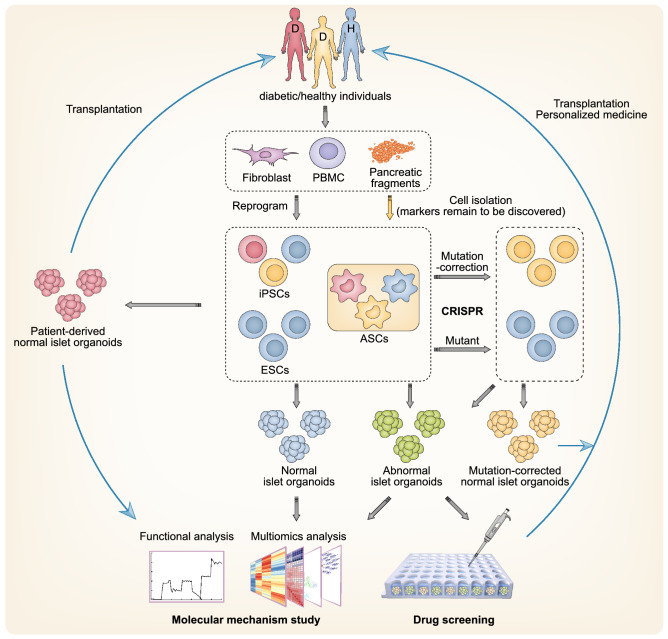
**Promising applications of islet organoids in disease modeling and personalized medicine**. Personalized islet organoids can be derived from either iPSCs reprogrammed from fibroblasts/peripheral blood mononuclear cells (PBMCs) or ASCs sorted from pancreatic fragments. Islet organoids derived from hESCs or iPSCs reprogrammed from healthy individuals are able to serve as a novel model system for human islet development and function research. Patient-derived iPSCs/ASCs can be used to generate functional islet organoids with or without mutation correction (Millman et al., 2016; Manzar et al., 2017; Saarimaki-Vire et al., 2017; Balboa et al., 2018; Ma et al., 2018; Maxwell et al., 2020), providing unlimited sources of islets for autologous transplantation. The abnormal islet organoids derived from patients or gene-edited hESCs faithfully recapitulate developmental and functional defects under pathological conditions, offering novel valuable platforms for the study of pathological mechanisms and personalized drug screening

As the developmental and functional properties of islet organoids have become more representative of physiological human islets, islet organoids can function as surrogates for inaccessible human islets, especially those from diabetic individuals. The combination of gene editing technologies, such as CRISPR/Cas9 and refined iPSC technology, allows the manipulation of islet organoids to investigate the developmental, functional, and pathological mechanisms of normal and diabetic human islets and to develop personalized and informative drug screening platforms (Fig. 2).

### Models for studying islet development

Although the mechanisms governing islet development are largely conserved between human and murine models, human-specific developmental events and structural or functional characteristics arise remarkable attention (Nair and Hebrok, 2015). Islet organoids derived from hPSCs are a promising system for investigating pivotal niche signals and biochemical or biophysical mechanisms governing islet development and developmental defects that result in congenital diabetes. In turn, this information can be further used to improve the design of differentiation strategies to enhance the maturation of islet organoids. Using sc-β cells, Sharon et al. found that WNT inhibition was a major regulatory mechanism in endocrine differentiation, capable of adjusting the induction output of β-like cells (Sharon et al., 2019). Adjusting the balance between proliferation and differentiation of pancreatic progenitors could expand the progenitor population to generate increased numbers of endocrine cells and force the differentiation of the pancreatic progenitors to eliminate undesired progenitors. However, effective strategies to manipulate this balance have not yet been developed. A possible solution is inhibition of YAP to manipulate the Hippo signaling pathway, whose activation facilitates pancreatic progenitor proliferation and whose inhibition favors endocrine specification (Rosado-Olivieri et al., 2019). Overexpression of YAP in β-like cells of derived islet organoids revealed a subpopulation of β cells with activated LIF pathways and increased proliferative capacity (Rosado-Olivieri et al., 2020), indicating the ability of these cells to reflect subtle characteristics of human islets.

Combined with gene editing technologies such as CRISPR/Cas9, organoids have the capacity to accomplish the incomplete gene regulatory network of islet development and pathogenesis. CRISPR screening in sc-β cells identified that the vitamin D receptor (VDR) is required for β cell differentiation and maintenance *in vitro*, and subsequent studies revealed an unrecognized role of VDR in β cell survival under inflammatory stress (Wei et al., 2018). Combining a novel gene editing platform termed iCRISPR and hPSC-directed differentiation, Zhu et al. confirmed the roles of conserved transcription factors in mice and humans, revealed the novel regulator RFX6 in the specification of pancreatic progenitors, and reported an intriguing haploinsufficiency requirement of PDX1 in endocrine differentiation (Zhu et al., 2016). Moreover, *NGN3*^−/−^ hPSC derivatives showed negligible functional endocrine specification in several independent studies (McGrath et al., 2015; Zhu et al., 2016), and some patients carrying *NGN3* mutations retained at least partial endocrine function. Using *NGN3*^−/−^ hPSC derivatives, Zhang et al. found that *NGN3* mutations affected protein instability and DNA binding capacity during pancreas development (Zhang et al., 2019). Another transcription factor, MAFB, is expressed in adult human β-cells but absent from the murine counterparts. Using CRISPR/Cas9 gene editing coupled with endocrine cell differentiation strategies, Russell et al. generated MAFB knockout hPSCs and revealed that MAFB is essential for human β-cell identity during endocrine cell development (Russell et al., 2020). Applying state-of-the-art strategies to establish 3D islet organoids may further systematically elucidate the developmental and functional mechanisms involving β cells and their regulatory interactions with non-β endocrine and nonendocrine cells.

### Models for disease research

Current model systems limit the research and exploration of diabetes and other disease mechanisms; for example, cell lines respond poorly to glucose stimulation and cannot model interactions between β cells and other cell types, which may play roles in islet function and the onset of diabetes. Islet organoids provide a novel system in which to study diabetes. β cells in islets are destroyed during disease progression in T1D and thus were previously difficult to be studied. Currently, iPSCs derived from patients with T1D are used to generate new models demonstrating that cells respond to different forms of β cell stress *in vitro* (Millman et al., 2016). Analysis of three T2D-related genes, *CDKAL1*, *KCNQ1*, and *KCNJ11*, which were identified by GWAS, in gene-edited hESCs showed normal endocrine specification but impaired insulin secretion *in vitro* and *in vivo* (Zeng et al., 2016). These phenotypes mimicked symptoms in patients carrying mutations in these genes, whereas animal models showed irrelevant phenotypes (Asahara et al., 2015). Wolfram syndrome, an autosomal recessive disease caused by mutations in *WFS1*, is characterized by juvenile-onset diabetes (Rohayem et al., 2011). β-like cells differentiated from iPSCs derived from Wolfram syndrome patients showed decreased insulin content and increased activity of molecules related to endoplasmic reticulum (ER) stress, indicating that β cell failure is caused by WFS1 deficiency (Shang et al., 2014; Maxwell et al., 2020). Congenital hyperinsulinism (CHI), a rare genetic disorder related to mutations in sulfonylurea receptor 1 (SUR1), encoded by *ABCC8*, is characterized by excessive insulin secretion and hypoglycemia (Nessa et al., 2015). *ABCC8-*deficient sc-β cells recapitulated the disease phenotypes of CHI *in vitro*, indicating that these cells could be an attractive model for further elucidating SUR1 function (Guo et al., 2017). We have summarized the disease models related to islet organoids and sc-β cells in Table 1.

**Table 1:** Representative studies establishing islet organoids as disease models and drug screening platforms

Genes	Related types of diabetes/Symptoms of patients	Derivation of islet organoids	Phenotypes of islet organoids	Drug/Chemical screening	References
Non-specific	T1D	Patients-derived	Resemble human islets; normal GSIS	Not mentioned	(Manzar et al., 2017)
Non-specific	T1D	Patients-derived	Respond to glucose *in vitro* and *in vivo*	Tolbutamide; Liraglutide; LY2608204	(Millman et al., 2016)
Non-specific	T1D	Patients-derived	T cell activation and killing when sc-β cells were co-cultured with autologous PBMCs	Not mentioned	(Leite et al., 2020)
*RNLS*	T1D	KO and WT hiPSCs	Resistance to thapsigargin-induced apoptosis	pargyline	(Cai et al., 2020)
*CDKAL1*	T2D	KO hESCs	Defective insulin secretion; hypersensitivity to glucolipotoxicity	T5224	(Zeng et al., 2016)
*GSTT1*	Increased risk to T2D	KO hESCs	Hypersensitive to propargite (a pesticide causing β cell death)	Not mentioned	(Zhou et al., 2018)
*KCNQ1*	T2D	KO hESCs	Defective insulin secretion	Not mentioned	(Zeng et al., 2016)
*KCNJ11*	T2D	KO hESCs	Defective insulin secretion	Not mentioned	(Zeng et al., 2016)
*SIX2*	T2D	KD and KO hESCs	Impaired GSIS	Not mentioned	(Velazco-Cruz et al., 2020)
*SLC30A8* (p.Arg138*)	Resistance to T2D	Mutant hiPSCs	Reduced formation of β like cells; SLC30A8 expression haploinsufficiency	Not mentioned	(Dwivedi et al., 2019)
*VDR*	T2D	KD hiPSCs	Increased cytokine-induced cell death	Cal + iBRD9	(Wei et al., 2018)
*HNF4A*	MODY1	Patients-derived	No specific phenotypes compared to hESC-derived β like cells	Not mentioned	(Vethe et al., 2017)
*HNF1A*	MODY3	KO hESCs	Impaired GSIS; α-cell preference in islet development	Not mentioned	(Cardenas-Diaz et al., 2019)
*HNF1B*	MODY5	Patients-derived	Retardation of cell growth; mutant mRNA degradation	Not mentioned	(Yabe et al., 2015)
*NEUROD1*	MODY6, ND	Mutant hESCs	Impaired β cell differentiation	Not mentioned	(Romer et al., 2019)
*GATA4*	ND and pancreatic agenesis	KO hESCs	Impaired pancreatic progenitor formation	Not mentioned	(Shi et al., 2017)
*GATA6*	ND and pancreatic agenesis	KO and mutant hESCs and hiPSCs	Haploinsufficient requirement of GATA6 in pancreatic progenitors and β cells development	Not mentioned	(Shi et al., 2017)
*GLIS3*	ND, T1D, T2D	KO hESCs	Impaired β cell formation; increased apoptosis	Galunisertib	(Amin et al., 2018)
*INS* *(mutation C96R, C109Y and ATG>ATA)*	ND	Patients-derived (with or without mutation correction)	ER stress; reduced proliferation of β cells; reduced insulin secretion and increased α cell mass *in vivo*; rescued by mutation correction	Not mentioned	(Balboa et al., 2018; Ma et al., 2018)
*NGN3*	ND (some patients)	KO hESCs and induced mutant expression in hESCs derivatives	No functional endocrine formation in KO hESCs, which was rescued by hypomorphic mutants.	Not mentioned	(McGrath et al., 2015; Zhu et al., 2016; Zhang et al., 2019)
*PDX1*	ND	KO hESCs	Haploinsufficient requirement for PDX1 in β cell differentiation	Not mentioned	(Zhu et al., 2016)
*RFX*	ND	KO hESCs	Impaired pancreatic progenitor formation	Not mentioned	(Zhu et al., 2016)
*STAT3*	ND and pancreatic hypoplasia	Patients-derived (with or without mutation correction)	Premature differentiation of pancreatic progenitors; pancreatic hypoplasia; increased α cell mass *in vivo*; rescued by mutation correction	Not mentioned	(Saarimaki-Vire et al., 2017)
*ABCC8*	Congenital hyperinsulinism and hypoglycemia	KO hESCs	Excess insulin secretion	Nifedipine, octreotide, nicorandil	(Guo et al., 2017)
*FOXA2*	Various types of diabetes	KO hESCs	Impaired pancreatic progenitor formation	Not mentioned	(Lee et al., 2019)
*WFS1*	Wolfram syndrome	Patients-derived (with or without mutation correction)	Decreased insulin content; ER stress; rescued by mutation correction	4PBA	(Shang et al., 2014; Maxwell et al., 2020)
*MAFB*	Not reported	KO hESCs	Preference commitment to δ or γ cells instead of α or β cells	Not mentioned	(Russell et al., 2020)

### Platforms for drug screening

Islet organoids are better platforms than other models for drug screening in diabetes. Via a chemical drug library, T5224 was discovered to be able to rescue β cell dysfunction in *CDKAL1*^−/−^ sc-β cells by inhibiting the FOS/JUN pathway, providing a potential treatment for T2D (Zeng et al., 2016). Induction of *GLIS3*^−/−^ hESCs showed impaired β cell differentiation and increased apoptosis. Moreover, a high-content chemical screening based on *GLIS3*^−/−^ hESCs derivatives identified a drug candidate, galunisertib, that could be used for treatment of *GLIS3*-associated diabetes (Amin et al., 2018).

With advances in iPSC technology and personalized medicine, patient-derived β cells and islet organoids can serve as informative disease models recapitulating the pathogenesis and phenotypes of specific patients and can be used to assess patient-specific drug reactions upon screening. In a proof-of-concept experiment to evaluate possible applications for drug screening, sc-β cells from T1D patient-derived hiPSCs treated with three antidiabetic compounds, tolbutamide, liraglutide and LY2608204, showed increased insulin release (Millman et al., 2016). Additionally, 4PBA successfully restored the glucose responsiveness of islet organoids derived from patients with Wolfram syndrome (Shang et al., 2014). However, the limited functionality and reproducibility of established islet organoids require validation in other systems.

### Models for studying infection with SARS-CoV-2 and other viruses

**Figure 3:**
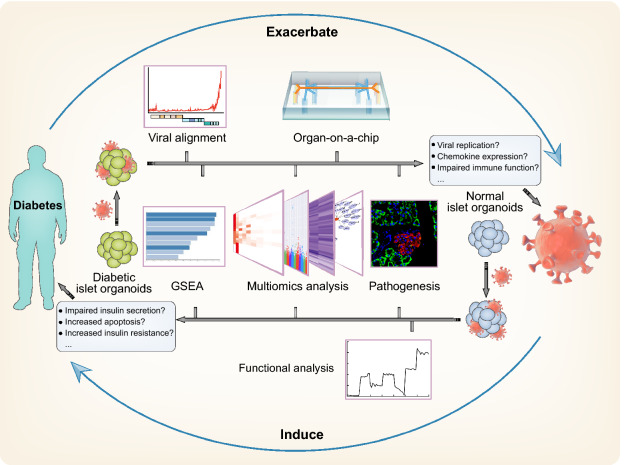
**Islet organoids as a promising model for SARS-CoV-2 infection**. Evaluation of SARS-CoV-2-infected diabetic islet organoids may elucidate the mechanisms underlying the elevated mortality in diabetic COVID-19 patients. Identification of islet dysfunction in SARS-CoV-2-infected normal islet organoids would help to clarify the onset of diabetes in COVID-19 patients

During the current worldwide COVID-19 pandemic, diabetes has been reported to be a high-risk factor for both SARS-CoV-2 infection and severe symptoms in infected patients (Muniyappa and Gubbi, 2020). Significantly increased mortality and comorbidities have been observed in patients with COVID-19 and pre-existing T1D and T2D, and these outcomes can be ameliorated with glycemic control (Zhu et al., 2020). On the other hand, COVID-19 can also induce or worsen hyperglycemia through unclear mechanisms (Bornstein et al., 2020). Hence, islet organoids have potential as a promising human model at the whole-organ level for revealing the mechanisms underlying putative SARS-CoV-2 infection and destruction of pancreatic islets, as well as for assessing therapeutic drugs for both treatment of COVID-19 and glycemic control in the COVID-19 context (Fig. 3).

As a proof-of-concept experiment, islet organoids and several other organoids were recently used to model SARS-CoV-2 infection in various human organs (Han et al., 2020; Lamers et al., 2020; Suzuki et al., 2020; Yang et al., 2020). hESC-derived sc-β and sc-α cells expressed the coronavirus spike protein receptor ACE2 and were permissive to SARS-CoV-2 infection with robust viral replication *in vitro* and *in vivo*, consistent with the results in human primary β and α cells, suggesting the authenticity of islet organoids in modeling virus infection (Yang et al., 2020). SARS-CoV-2-infected sc-β and sc-α cells showed increased insulin resistance and chemokine expression and a decrease in endocrine functional pathways due to increased apoptosis, resulting in insulin deficiency and even diabetes onset in some COVID-19 patients (Yang et al., 2020). However, the expression of ACE2 is relatively low in endocrine compartments compared with exocrine compartments. Evidence has shown that pancreatic ducts and microvasculature are more likely targets in SARS-CoV-2 infection (Coate et al., 2020; Kusmartseva et al., 2020). Therefore, whether islet organoids are an ideal SARS-CoV-2 infection model remains to be observed and determined. As a safe and convenient system, organ-on-a-chip technology can identify the crosstalk between different SARS-CoV-2-infected organs and elucidate the mechanisms underlying the elevated mortality in diabetic COVID-19 patients.

In addition to SARS-CoV-2, several other viruses are involved in the onset of T1D (Op de Beeck and Eizirik, 2016), and the islet organoid system has shown the capacity to model both virus infection and endocrine cell dysfunction, offering cellular and molecular insights into human-relevant pathogenesis.

### Potential organ sources for tissue replacement and personalized therapy

Islet organoids have the potential to overcome the shortage of cadaveric islet donors by offering an unlimited number of functional islets on demand for regenerative therapy. The combination of iPSC and CRISPR technologies makes it possible to derive patient-personalized islet organoids with or without gene correction (Shang et al., 2014; Millman et al., 2016; Saarimaki-Vire et al., 2017; Balboa et al., 2018), paving the way for autologous transplantation and minimizing immune rejection. Compatibility of these methods with novel technology such as PD-L1 generated immune-evasive islet organoids which allows them to offer a new therapeutic strategy for autoimmune diabetes, like T1D (Yoshihara et al., 2020). Recently, phase I/II clinical trials for subcutaneous implantation of hESC-derived pancreatic endodermal or progenitor cells, which will further mature into functional endocrine cells *in vivo*, into T1D patients were approved (Viacyte, 2014, 2016, 2017a, b, 2019). Different kinds of encapsulation devices have been applied to contain and protect the implanted cells (Cooper-Jones and Ford, 2016). The PEC-Encap (VC-01) prevents immune cell accession from direct contact with the implanted cells, but allows vital nutrients and proteins to travel between the inside cells and the outside blood vessels (Agulnick et al., 2015). The PEC-Direct (VC-02) allows blood vessels to enter the device and directly interact with the implanted cells, while immune cells are also capable of entering the device (Cooper-Jones and Ford, 2016).

## REMAINING CHALLENGES AND PROSPECTIVE SOLUTIONS

Although improvements have allowed 3D hormone-producing islet organoids to partially reproduce islet morphology and function, certain limitations need to be addressed.

### Challenges in the establishment of islet organoids

#### Immaturity

Generally, established islet organoids do not fully recapitulate the behavior of native islets at the molecular and functional levels; for example, they have poor insulin secretion ability and lack dynamic insulin release. To improve the performance of islet organoids, one of the key fundamental efforts is characterization of the discrepancies between derived islet organoids and native islets, as well as updating induction strategies. Multiomics analysis approaches, especially single-cell RNA sequencing (scRNA-seq), have widely been used and shown to be informative for revealing molecular defects hindering the maturation and application of islet organoids. The results showed that most derived islet organoids rarely express certain mature β cell transcription factors, including MAFA, UCN3 and SIX3, differently express mature β cell functional genes, including PCSK1,PCSK2,CPE (Suckale and Solimena, 2010; Wang et al., 2011; Li et al., 2018), and the ratio of major endocrine cells is much different from that of native islets (Bakhti et al., 2019; Veres et al., 2019; Augsornworawat et al., 2020). Real-time imaging of the mouse embryonic pancreas demonstrated how molecular interactions and organ architecture influence the cell fate specification of pancreatic progenitors to commit to endocrine differentiation (Nyeng et al., 2019). Similar studies on islet organoids or native human islets will substantially contribute to the current understanding of islet development.

#### Heterogeneity

Currently established islet organoids have lost at least part of the heterogeneity compared with their *in vivo* counterparts. Although most islet organoids contain all or several of the five major islet cell types, their proportion of β cells is much higher than that of native human islets. Human islets contain more non-β endocrine cells, which disperse more widely and without a prominent core, than rodent islets (Cabrera et al., 2006; Arrojo e Drigo et al., 2015; Roscioni et al., 2016). In addition, β cells are highly heterogeneous and comprise several subpopulations differing in electrical activity, glucose responsiveness, and proliferative activity (Meda et al., 1984; de Vargas et al., 1997; Van de Casteele et al., 2013; Bader et al., 2016; Roscioni et al., 2016). The mechanism and biological relevance of such heterogeneity in humans are not fully understood, and high-fidelity islet organoids may help elucidate this issue. Nonendocrine supporting cells, including endothelial cells and neurons, are also barely observed in current islet organoids. These supporting cells are essential components that form vascular and innervation networks, which facilitate islet development by integrating islets into the circulatory and nervous systems (Ahren, 2000; Ranjan et al., 2009; Borden et al., 2013). Incorporation of supporting cells in islet organoids may not only improve the maturity and functionality of the derived islet organoids but also contribute to identifying the interactions between different cell types in human islets. Vascularized islet organoids have been attempted by several elaborate strategies (summarized above); however, innervated islet organoids still need to be pursued in the future.

Islet organoids have certain undesired heterogeneity. ScRNA-seq revealed that most derived islet organoids comprise large numbers of proliferating progenitors or precursors and irrelevant or uncharacterized cell types (Veres et al., 2019; Weng et al., 2020), which hinders their *in vitro* application and *in vivo* transplantation. Elimination of such undesired heterogeneity requires either improvement of the induction efficiency or cell isolation to remove the unwanted populations and enrich the endocrine populations. CD49a has been identified as a surface marker for sc-β cells and used to purify β-like cells (Veres et al., 2019). GP2 has been determined to be a surface marker for PDX1^+^/NKX6.1^+^ pancreatic progenitors. This protein has been employed to enrich PDX1^+^/NKX6.1^+^ cells, eliminate ~20%–40% of undifferentiated hPSCs, early derivatives or irrelevant cell types, and generate a higher percentage of β- and α-like cells (Ameri et al., 2017; Cogger et al., 2017). Other innovative strategies to remove the undesired products have been introduced to avoid teratoma formation after transplantation and to indirectly elevate induction efficiency. Insertion of the suicide gene *iC9* in the *SOX2* locus enabled induction of apoptosis of undifferentiated hPSCs upon treatment of the final-stage products with the iC9 inducer AP1903, while the differentiated hPSC derivatives were unaffected (Wu et al., 2019).

#### Batch-to-batch variations

Batch-to-batch variations in the efficiency of differentiation, organoid quality (e.g., size and shape), and organoid viability or functionality are significant. This stochasticity reflects the absence of pivotal niche signals in the culture cocktail; thus, a complete and detailed understanding of the islet development process is required to optimize growth factor cocktail recipes. Additionally, cell-specific subtle modifications of induction strategies are needed, especially for patient-derived iPSCs. iPSCs derived from patients with T1D lost resistance to differentiation after transient demethylation treatment (Manzar et al., 2017). The demethylation-treated sc-β cells displayed no significant disparities compared with sc-β cells derived from healthy individuals, highlighting the necessity of cell-specific induction strategies (Manzar et al., 2017).

### Challenges in the clinical applications of islet organoids

Islet organoids have shown potential as an organ source for the treatment of diabetes. However, several issues related to islet organoid transplantation must be carefully addressed.

#### Tumorigenicity

Although iPSCs show promise for personalized therapy, their issues with clinical safety remain to be addressed. The incorporation of stem cell derivatives in the human body raises concerns with respect to tumors (Zhu and Fan, 2018), especially teratoma formation, which is further exacerbated by the presence of uncharacterized proliferative cells in established organoids. The propensity for teratoma formation from iPSC derivatives has been reported to be affected by many factors, including iPSC tissue origin and strategies used for reprogramming, differentiation or transplantation (Miura et al., 2009). For example, c-Myc, an indispensable transcription factor in the reprogramming cocktail, is an oncogene (Okita et al., 2007). If it is omitted during the reprogramming, iPSCs would be generated with low efficiency (Nakagawa et al., 2010). Thus, prudent selection of induction strategies and rigorous examination before transplantation are indispensable.

#### Low survival and immunogenicity

Low survival and limited duration of euglycemia after transplantation have hindered transplantation of both donor islets to patients and islet organoids to mice. Low vascularization and immune rejection have accounted for significant islet cell loss. To overcome these issues, scientists have proposed elaborate encapsulation devices to shelter organoids from the immune system. Embedding a catheter into subcutaneous tissues at the transplant site in host mice before islet implantation can generate a vascularized space (Pepper et al., 2015). In addition, a recent study proposed using CRISPR/Cas9 to reduce the immunogenicity of stem cells by eliminating the expression of human leukocyte antigen (HLA) (Han et al., 2019).

#### Undefined ingredients and contamination

Most established islet organoids rely on Matrigel. However, Matrigel is not an approved material for clinical applications due to the presence of undefined ingredients. Some groups also use mouse embryonic fibroblasts (MEFs) as feeders for hPSC passaging or fetal bovine serum (FBS) during differentiation, thus resulting in xenogeneic contamination. The influence of xenogeneic contamination and undefined ingredients on the cellular microenvironment and organoid morphology or function remains unclear. To render the induced products amenable to transplantation, organoids need to be fully purified by enzymatic digestion to remove scaffolds (Wang et al., 2017; Rossen et al., 2020). Another strategy is culture of the organoids in medical grade scaffolds, either derived from decellularized porcine tissues (Giobbe et al., 2019) or synthesized with defined components. Various kinds of hydrogels, including PEG-based hydrogels functionalized with laminin (Greggio et al., 2013), PLG scaffolds containing exendin-4 microparticles (Kasputis et al., 2018), and electrospun PLLA/PVA scaffolds (Ojaghi et al., 2019), have been developed to facilitate islet or β cell specification and maturation, substantially accelerating the clinical applications of islet organoids.

## SUMMARY AND PERSPECTIVES

Islet organoids enable scientists to transform the understanding and treatment of diabetes. An ideal islet organoid should recapitulate the features of islet development and function of mature islets under physiological and pathological conditions. To improve the performance of current islet organoids, a more detailed understanding of islet development is necessary, and interdisciplinary methods, including organ-on-a-chip, 3D scaffold or bioprinting, are welcomed to construct more appropriate physiological niches for islet organoid development. As a highly expected application, islet organoids are considered an unlimited organ resource for transplantation and the treatment of diabetes, although several issues and safety concerns regarding organoid transplantation remain to be addressed. Organ transplantation techniques dedicated to reducing host immune rejection are also evolving (O’Sullivan et al., 2011; Pathak et al., 2019). The development of islet organoids will be a milestone in understanding and treating diabetes.

## Abbreviations

2D: two-dimensional
3D: three-dimensional
ACE2: angiotensin-converting enzyme 2
ASC: adult stem cells
ATP: adenosine triphosphate
BMP: bone morphogenetic protein
Cas9: CRISPR-associated protein 9
CHI: congenital hyperinsulinism
COVID-19: coronavirus disease 2019
CRISPR: clustered regularly interspaced short palindromic repeats
DE: definitive endoderm
EB: embryonic bodies
ECC: endocrine cell clusters
ECM: extracellular matrix
EP: endocrine precursors
ER: endoplasmic reticulum
ESC: embryonic stem cells
FBS: fetal bovine serum
GCG: glucagon
GHRL: ghrelin
GSEA: gene set enrichment analysis
GSIS: glucose-stimulated insulin secretion
GWAS: genome-wide association studies
HLA: human leukocyte antigen
HUVEC: human umbilical vein endothelial cells
iC9: inducible caspsae-9
INS: insulin
iPSC: induced pluripotent stem cells
LIF: leukemia inhibitory factor
MEF: mouse embryonic fibroblast
MODY: maturity-onset diabetes of the young
MSC: mesenchymal stem cells
ND: neonatal diabetes
PBMC: peripheral blood mononuclear cell
PdBU: phorbol 12,13-dibutyrate
PEG: polyethylene glycol
PEGDE: polyethyleneglycol diglycidyl ether
PGK: phosphoglycerate kinase
PKC: protein kinase C
PLG: polylactide-co-glycolide
PLLA/PVA: poly-l-lactic acid/polyvinyl alcohol
PP: pancreatic progenitors
PPY: pancreatic polypeptide
PSC: pluripotent stem cells
SARS-CoV-2: severe acute respiratory syndrome coronavirus 2
sc-β: stem-cell-derived β cells
scRNA-seq: single-cell RNA sequencing
SST: somatostatin
SUR1: sulfonylurea receptor 1
T1D: type 1 diabetes
T2D: type 2 diabetes
TALEN: transcription activator-like effector nucleases
TGF: transforming growth factor
VDR: vitamin D receptor
VEGFA: vascular endothelial growth factor A
